# MiR-542-3p Suppresses Neuroblastoma Cell Proliferation and Invasion by Downregulation of KDM1A and ZNF346

**DOI:** 10.1515/biol-2020-0018

**Published:** 2020-04-10

**Authors:** Qiang Wei, Zhao Guo, Dong Chen, Xinjian Jia

**Affiliations:** 1 Department II of General Surgery, Xi'an Children's Hospital, Xi'an, Shaanxi, China

**Keywords:** MiR-542-3p, KDM1A, ZNF346, Proliferation, Invasion, Neuroblastoma

## Abstract

Neuroblastoma is one of the most common malignancies in infants and children. MicroRNAs (miRNAs) have been reported as significant regulators that play important roles in neuroblastoma development. This research aimed to analyze the functional mechanism of miR-542-3p in neuroblastoma. Here, we found that miR-542-3p was downregulated and KDM1A as well as ZNF346 were upregulated in neuroblastoma tissues and cells. Both overexpression of miR-542-3p and the knockdown of KDM1A suppressed cell proliferation and invasion in neuroblastomas. Moreover, miR-542-3p reduced the levels of KDM1A and ZNF346 through interaction. Both KDM1A overexpression and ZNF346 upregulation weakened the effect of miR-542-3p on neuroblastoma cells. Besides, miR-542-3p negatively regulated tumor growth *in vivo*. Our results suggested that miR-542-3p suppressed cell proliferation and invasion by targeting KDM1A and ZNF346 in neuroblastomas, providing a theoretical basis for the treatment of neuroblastoma.

## 1 Introduction

Neuroblastoma, a malignancy in children, is derive from the primitive cells of the sympathetic nervous system during embryonic development [1, 2]. Its clinic symptoms depend on the location of the tumor [3]. According to the statistics, neuroblastomas account for approximately 15% of all childhood cancer-related deaths due to their aggressive nature and diagnostic difficulty, despite it consisting of only 8% of childhood malignancies [4, 5]. In recent years, molecular techniques have been used for the study of clinical neuroblastoma, but the treatment of patients remains unsatisfactory. Therefore, it is imperative to identify new molecular mechanisms for the therapy of neuroblastoma.

MicroRNAs (miRNAs), with 19-22 nucleotides, are non-coding RNAs that are involved in post-transcriptional regulation or degradation of the mRNA by targeting the 3’-untranslated region (3’-UTR) of mRNA [6]. Present evidence suggests that genes, regulated by miRNAs, are related to cell proliferation, apoptosis, and invasion in human cancers [7, 8]. Nowadays, miRNAs are considered as potential biomarkers for disease diagnosis [9]. MiR-542-3p, an endogenous miRNA, is one of the miR-542 isoforms that have low expression level in cancer cells. It was reported that miR-542-3p suppressed tumor growth in a variety of human cancers, including colorectal cancer [10], colon cancer [11], melanoma [12], and epithelial ovarian cancer [13]. miR-542-3p also inhibited tumor growth through downregulation of surviving in neuroblastoma [14]. However, whether miR-542-3p regulating tumor growth through other pathways remains unknown in neuroblastoma.

Lysine-specific demethylase 1A (KDM1A), also known as LSD1, is the first identified enzyme that exerts function in histone demethylation [15]. *In vivo*, KDM1A interacts with substrates through its SWIRM domain and demethylates it in the presence of REST corepressor (CoREST), which regulates expression of downstream genes under both physiological and pathological conditions [16, 17]. Present evidence suggests that KDM1A was highly expressed in various human cancers such as chondrosarcoma, rhabdomyosarcoma, neuroblastoma, prostate, bladder, breast, colorectal, gastric, and lung cancer [18, 19, 20]. In these cancer cells, KDM1A generally plays a positive role for their growth. For example, KDM1A promotes cell invasion through silencing the TIMP metallopeptidase inhibitor 3 (TIMP3) in non-small lung cancer [21], inhibits cell apoptosis in glioma [22], and promotes cell proliferation in neuroblastoma [23]. Zinc finger protein 346 (ZNF346), a member of ZNF protein family, is reported to be related to human diseases [24, 25]. In neuroblastoma, ZNF346 is reported to modulate cell proliferation and apoptosis [26]. This data means that KDM1A and ZNF346 are important for development of cancer cells, so it is essential to study the function of KDM1A and ZNF346 for the understanding of the molecular mechanism of cancer cell growth.

Here, we found that KDM1A and ZNF346 were two potential targets of miR-542-3p. Then, the levels of miR-542-3p, KDM1A, and ZNF346 in neuroblastoma tissues and cells was explored. Next, their functions and association were investigated. Furthermore, a new mechanism that miR-542-3p affecting neuroblastoma cell growth through modulating KDM1A and ZNF346 levels was confirmed.

## 2 Materials and Methods

### 2.1 Tissue samples and Cell culture

A total of 37 neuroblastoma tissues and corresponding normal tissues from the patients at the hospital of Xi’an Children’s Hospital were collected and stored at -80°C.

Two human neuroblastoma cell lines SK-N-SH (ATCC® HTB-11™) as well as SK-N-AS (ATCC® CRL-2137™) and one normal cell line HUVEC (ATCC® CRL-1730™) were purchased from the American Tissue Culture Collection (ATCC, Manassas, VA, USA). All cells were grown on Dulbecco’s Modified Eagle Medium (DMEM; Invitrogen, Carlsbad, CA, USA) supplemented with 10% fetal bovine serum (FBS; Thermo Fisher Scientific, Waltham, MA, USA) at 37°C in an incubator with 5% CO_2_.

**Informed consent**: Informed consent has been obtained from all individuals included in this study.

**Ethical approval**: The research related to human use has been complied with all the relevant national regulations, institutional policies and in accordance the tenets of the Helsinki Declaration, and has been approved by the Ethics Committee of Xi’an Children’s Hospital.

### 2.2 Plasmids and cell transfection

MiR-542-3p mimic and miR-NC were synthesized by GenePharma (Shanghai, China). Small interfering RNA against KDM1A (si-KDM1A) was synthesized by Ribobio Inc (Guangzhou, China), the sequences for the si-KDM1A were as follows: sense, 5’-GCUGCAGGAUCAUCUGGAAdTdT-3’ and antisense, 5’-UUCCAGAUGAUCCUGCAGCdTdT-3’. The PCR fragment amplified by a pair of primer (forward primer, 5’-CGGAATTCGGCGGCCCGAGATGTTAT-3’ and reverse primer, 5’-CCCTCGAGTGGGCCTCTTCCCTTAGAAT-3’) was cloned into pcDNA3.1 vector to generate pcDNA3.1-KDM1A. Human cDNA was used to amplify the 3’-UTR of KDM1A mRNA using the following primers: forward primer, 5’-CCCTCGAGGCACAGGGAGGAACTT-3’ and reverse primer, 5’-TTGCGGCCGCACAAAAACCCCACACACC-3’, the PCR fragments were inserted into psiCHECK2 vector, and mutation in miR-542-3p binding sites was carried out using a fast mutation kit (Santa Clara, CA, USA). Similarly, si-ZNF346 was purchased from GenePharma (Shanghai, China). ZNF346 full length was inserted into pcDNA3.1 vector to construct the ZNF346 overexpression vector, 3’-UTR of ZNF346 mRNA or its mutant was cloned into psiCHECK2 vector to perform the dual luciferase reporter assay.

Transfection assay was performed using Lipofectamine 2000 transfection reagent (Invitrogen) according to the manufacturer’ instruction. The groups used in this study were as follows: miR-NC, miR-542-3p, si-NC, si-KDM1A, miR-NC + KDM1A/ZNF346 3’-UTR-WT, miR-NC + KDM1A/ ZNF346 3’-UTR-MUT, miR-542-3p + KDM1A/ZNF346 3’-UTR-WT, miR-542-3p + KDM1A/ZNF346 3’-UTR-MUT, miR-542-3p + pcDNA3.1, miR-542-3p + pcDNA3.1-KDM1A/ZNF346.

### 2.3 RNA extraction and quantitative real-time polymerase chain reaction (qRT-PCR)

TRIzol (Invitrogen) was used to extract the total RNA from neuroblastoma tissues or cells according to the manufacturer’s protocol. RNA concentration was determined before the reverse transcription reaction that was performed using a Prime Script RT reagent Kit (Takara, Dalian, China). QRT-PCR was carried out with SYBR green (Applied Biosystems, Foster City, CA, USA). U6 snRNA and glyceraldehyde 3-phosphate dehydrogenase (GAPDH) were used as reference genes for the relative expression of miR-542-3p, KDM1A, and ZNF346 calculated using the 2^-ΔΔCt^ method. The condition of the RCR action was as follows: 95°C for 3 min, 40 cycles of 95°C for 10 s, and 58°C for 30 s. The primes were listed in table 2.

**Table 1:** Analysis of the correlation between expression of miR-542-3p and clinicopathological parametersin in NB patients

Variable	Patients,n	miR-542-3p expression		P-value
		Low	High	
Age,years	37	22	15	0.326
<18 months	18	11	7	
≥18 months	19	11	8	
Sex				0.289
Male	15	9	6	
Female	22	13	9	
N-myc status				0.334
Amplification	20	11	9	
Unamplification	17	11	6	
Clinical stage of INSS				0.021
I-II	12	7	5	
III-IV	25	15	10	

**Table 2:** Primer sequences of genes

Gene	Primers	sequence
MiR-542-3p	Forward	5’-GCCGCAAAGTGCTTACAGTG-3’
	Reverse	5’-TGCAGGGTCCGAGGTAT-3’
KDM1A	Forward	5’-ATCTGCAGTCCAAAGGATGG-3’
	Reverse	5’-GCCAACAATCACATCGTCAC-3’
ZNF346	Forward	5’-TCGTGGGAAACCTTAGAAGCGG-3’
	Reverse	5’-GCAGCACTGTTGACATGGTCTG-3’
U6	Forward	5’-TGCGGGTGCTCGCTTCGGCAGC-3’
	Reverse	5’-CCAGTGCAGGGTCCGAGGT-3’
GAPDH	Forward	5’-CCGGGAAACTGTGGCGTGATGG-3’
	Reverse	5’-AGGTGGAGGAGTGGGTGTCGCTGTT-3’

### 2.4 Western blot assay

Total proteins were isolated from cells using RIPA buffer (Beyotime Biotechnology, Shanghai, China), separated by dodecyl sulfate, sodium salt-Polyacrylamide gel electrophoresis (SDS-PAGE, 10%), and transferred onto polyvinylidene didluoride (PVDF) membranes (Millipore, Billerica, MA, USA). The membranes were blocked by 5% skimmed milk at room temperature for 1 h, followed by incubation with primary antibodies (1: 1,000) at 4°C overnight. After washing with a TBST (Tris-buffered saline containing 0.1% Tween-20) buffer three times, the membranes were incubated with HRP-linked secondary antibodies (1:2,000) at room temperature for 1 h and then washed with a TBST buffer for five times. The proteins were visualized by Amersham ECL Plus (Amersham, Arlington Heights, IL, USA) and detected with UVchem Detection Device (Biometra, Gottingen, Germany). Primary antibodies against KDM1A, ZNF346, or β-actin (Abcam, Cambridge, MA, USA) were used in this study.

### 2.5 Cell proliferation and invasion assay

Cell proliferation was assessed by 3-(4, 5-dimethyl-2-thiazolyl)-2, 5-diphenyl-2-H-tetrazolium bromide (MTT) assay. After transfection, the cells were incubated for 0 h, 24 h, 48 h, or 72 h. Then, 20 μL MTT solution (5 mg/ mL, GD-Y1317, Shguduo Biomart Inc., Shanghai, China) was applied to treat 1 x 10^4^ cells for 4 h at 37°C. After the medium was removed, 150 μL dimethyl sulfoxide (DMSO; Sigma, St. Louis, MO, USA) was added into the cells for dissolution of the formazan product. Finally, the cell absorbance was measured at 490 nm using a microplate reader (Bio-Rad, Richmond, CA, USA).

The transwell chamber pre-coated with Matrigel (BD Biosciences, Franklin Lakes, NJ, USA) was prepared for the detection of cell invasion ability. After transfection, the cells were incubated for 16 h. 100 μL of serum-free medium containing 1× 10^6^ cells was added into the upper chamber and 500 μL cell medium with 10% FBS was added into the lower chamber. Crystal violet was used to stain the cells after the cells were incubated at 37°C for 12 h. Finally, the number of invasive cells was analyzed using an inverted microscope (magnification, x40; Olympus Corporation, Tokyo, Japan).

### 2.6 The dual luciferase reporter assay

The potential target genes and sites of miR-542-3p were predicted using the online tool DIANA (http://diana.imis.athena-innovation.gr/DianaTools/index.php). The 3’-UTR sequence of wide type KDW1A/ZNF346 (KDM1A/ZNF346 3’-UTR-WT) and its mutant (KDM1A/ZNF346 3’-UTR-MUT) containing mutant binding sites of miR-542-3p were cloned into a reporter vector, then the reporter vector and miR-542-3p or miR-NC were co-transfected into 5 x 10^4^ SK-N-SH and SK-N-AS cells. After incubation for 48 h, the luciferase activity was determined by the Lmax multiwall luminometer (Molecular Devices, LLC, Sunnyvale, CA, USA).

### 2.7 RNA immunoprecipitation (RIP) assay

RIP assay was performed with a Magna RNA immunoprecipitation kit (Millipore) according to manufacturer’s instructions. In brief, the cells were lysed by RIP buffer containing magnetic beads conjugated with Ago2 or IgG antibody after transfection with miR-NC or miR-542-3b for 48 h. TRIzol reagent was used to isolate immunoprecipitated RNAs. Finally, enrichment of KDW1A or ZNF346 was assessed by qRT-PCR assay.

### 2.8 Mouse xenografts

Firstly, 6-week-old female mice were injected with 1×10^6^ cells transfected with an empty vector or GV235-miR-542-3p-puromycin. Then, tumor volume (length × width × width/2) was measured every 7 d. 28 d later, the mice were sacrificed, and the tumor weight was analyzed.

**Ethical approval**: The research related to animals use has been complied with all the relevant national regulations and institutional policies for the care and use of animals. All animal experiments were approved by the Animal Research Committee of Xi’an Children’s Hospital.

### 2.9 Statistical analysis

All data were expressed as a mean ± standard deviation (SD) of at least three independent experiments. Statistical analysis was carried out by SPSS 22.0 software. *P*<0.05 was considered to be statistical significant.

## 3 Results

### 3.1 The expression levels of miR-542-3p, KDM1A, and ZNF346 were changed in neuroblastoma tissues and cells

**Figure 1:**
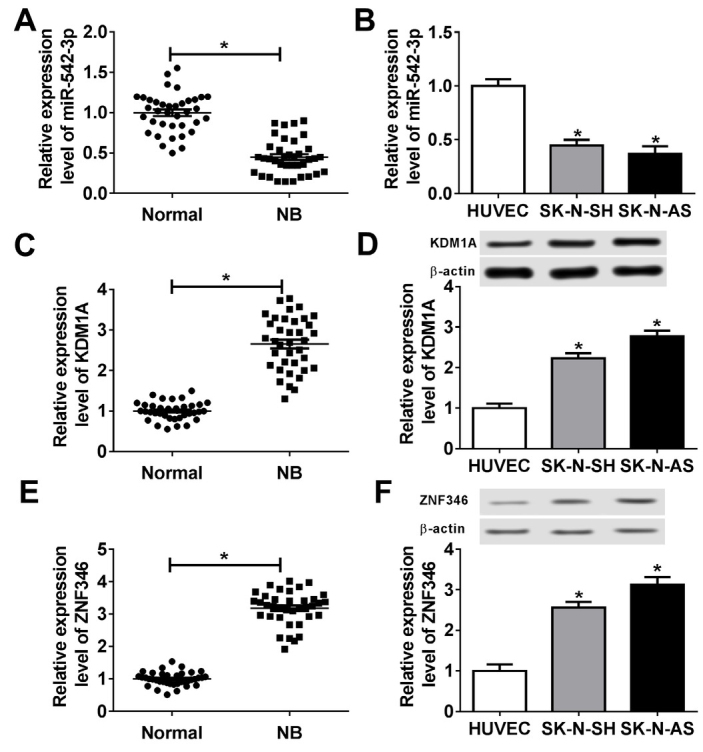
**The expression level of miR-542-3p, KDM1A, and ZNF346 in neuroblastoma tissues and cells**. (A and B) QRT-PCR assay was performed to detect the level of miR-542-3p in neuroblastoma tumor tissues (n=23) and matched normal tissues (n=23) (A) as well as in neuroblastoma cells (SK-N-SH and SK-N-AS) and normal cell lines (HUVEC) (B). (C and D) The transcription level of KDM1A in neuroblastoma tumor tissues (n=23) (C) and the protein level of KDM1A in neuroblastoma cells (SK-N-SH and SK-N-AS) (D) were determined by qRT-PCR assay and western blot assay, respectively. (E and F) The transcription level of ZNF346 in neuroblastoma tumor tissues (n=23) (E) and the protein level of ZNF346 in neuroblastoma cells (SK-N-SH and SK-N-AS) (F) were examined. *P<0.05.

Firstly, the levels of miR-542-3p, KDM1A and ZNF346 were determined in neuroblastoma. The results suggested that the expression level of miR-542-3p was significantly decreased in neuroblastoma tissues compared with matched adjacent normal tissues (Figure 1A), and was lower in neuroblastoma cells (SK-N-SH and SK-N-AS) than that in normal cells (HUVEC) (Figure 1B). Whereas the levels of KDM1A and ZNF346 were increased in neuroblastoma tissues/cells compared to that in normal tissues/cells (Figure 1C-F). From these data, it was possible that miR-542-3p acted as a tumor suppressor, and KDM1A as well as ZNF346 exerted oncogenic function.

### 3.2 The overexpression of miR-542-3p suppressed proliferation and invasion in neuroblastoma cells

**Figure 2:**
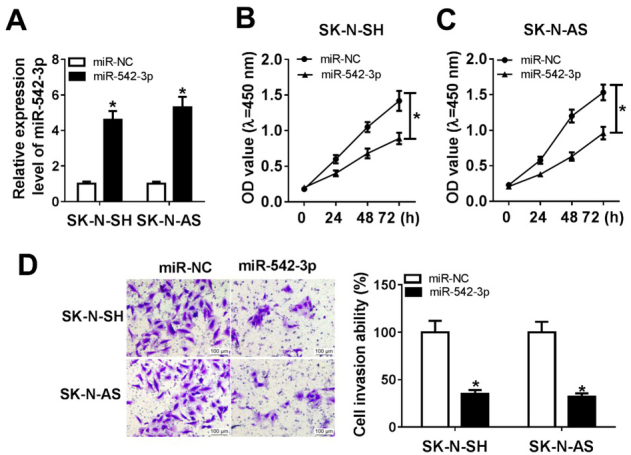
**The upregulation of miR-542-3p suppressed cell viability in neuroblastoma**. (A) The expression of miR-542-3p in cells transfected with miR-NC or miR-542-3p was detected by qRT-PCR assay. (B and C) MTT assay was used to assess cell proliferation in SK-N-SH cells (B) and SK-N-AS cells (C) after transfection of miR-542-3p. (D) Transwell assay was carried out to measure cell invasion in SK-N-SH and SK-N-AS cells transfected with miR-542-3p (D). *P<0.05.

To further analyze the effect of miR-542-3p on neuroblastoma cells, we transfected miR-542-3p into neuroblastoma cells (SK-N-SH and SK-N-AS) and examined its expression level. Analysis of qRT-PCR demonstrated that transfection with miR-542-3p increased miR-542-3p level (Figure 2A). Next, we determined cell viability by MTT assay and transwell assay, and found that the enforced expression of miR-542-3p significantly inhibited proliferation (Figure 2B and C) and invasion (Figure 2D) in both SK-N-SH and SK-N-AS cells. These data indicated that the overexpression of miR-542-3p suppressed cell proliferation and invasion in neuroblastoma *in vitro*.

### 3.3 The knockdown of KDM1A inhibited neuroblastoma cell proliferation and invasion

**Figure 3:**
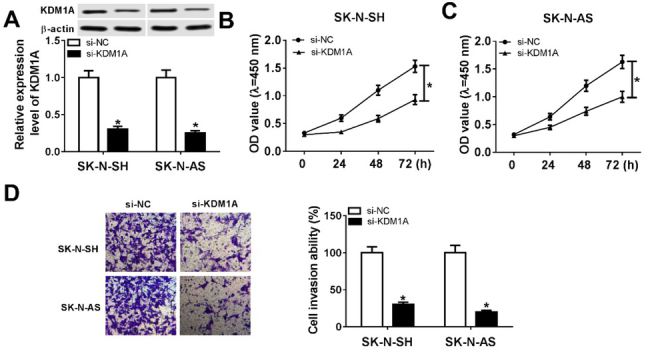
**The knockdown of KDM1A suppressed cell viability in neuroblastoma**. (A) QRT-PCR assay was performed to detect the level of miR-542-3p in SK-N-SH and SK-N-AS cells transfected with si-KDM1A or si-NC. (B and C) MTT assay was used to assess cell proliferation in SK-N-SH cells (B) and SK-N-AS cells (C) after transfection with si-KDM1A or si-NC. (D) Transwell assay was carried out to determine cell invasion in SK-N-SH and SK-N-AS cells transfected with si-KDM1A or si-NC. *P<0.05.

We also explored the effect of KDM1A on neuroblastomas. Firstly, SK-N-SH and SK-N-AS cells were transfected with si-NC or si-KDM1A. Then western blot assay showed that transfection with si-KDM1A dramatically decreased the protein level of KDM1A (Figure 3A). Then, MTT assay was performed to detect cell proliferation. As shown in Figure 3B and C, the knockdown of KDM1A significantly inhibited proliferation of SK-N-SH and SK-N-AS cells. In addition, the transwell assay demonstrated that the cells transfected with si-KDM1A exhibited lower invasive ability than the cells transfected with si-NC in neuroblastoma (Figure 3D). These results indicated that KDM1A knockdown suppressed cell proliferation and invasion in neuroblastoma *in vitro*.

### 3.4 MiR-542-3p reduced KDM1A expression through targeting KDM1A

**Figure 4:**
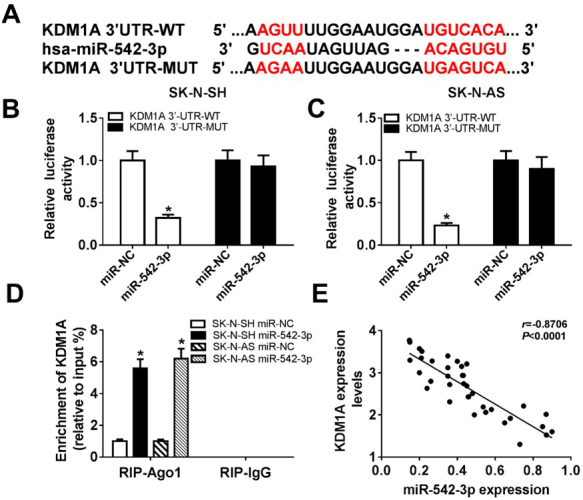
**The relationship between miR-542-3p and KDM1A in neuroblastoma cells**. (A) Prediction of miR-542-3p binding sites in the 3’-UTR of KDM1A by DIANA tool. (B and C) The dual luciferase reporter assay was performed to verify interaction between miR-542-3p and KDM1A in SK-N-SH (B) and SK-N-AS (C) cells co-transfected with miR-542-3p and KDM1A 3’-UTR-WT or KDM1A 3’-UTR-MUT1, miR-NC was used as a negative control. (D) The enrichment of KDM1A was measured in SK-N-SH and SK-N-AS cells transfected with miR-542-3p or miR-NC after RIP. (E) The association between miR-542-3p level and KDM1A level was explored. *P<0.05.

To explore the association between miR-542-3p and KDM1A, bioinformatics analysis website DIANA was used to predict the interaction between miR-542-3p and KDM1A. The results indicated that KDM1A was a potential target gene of miR-542-3p (Figure 3A). Then, the dual luciferase reporter assay was performed to verify this interaction. The analysis of luciferase activity suggested that luciferase activity was decreased in cells transfected with KDM1A 3’-UTR-WT and miR-542-3p, but not KDM1A 3’-UTR-MUT and miR-542-3p (Figure 4B and C), confirming interaction between miR-542-3p and KDM1A. Besides, RIP assay also confirmed that miR-542-3p interacted with KDM1A in SK-N-SH and SK-N-AS cells (Figure 4D).

Meanwhile, we also examined the relationship between the miR-542-3p level and the KDM1A level. As shown in Figure 4E, the KDM1A level was negatively correlated with miR-542-3p level. Thus, miR-542-3p repressed KDM1A expression by targeting KDM1A.

### 3.5 The upregulation of KDM1A reversed effect of miR-542-3p on neuroblastoma cells

**Figure 5:**
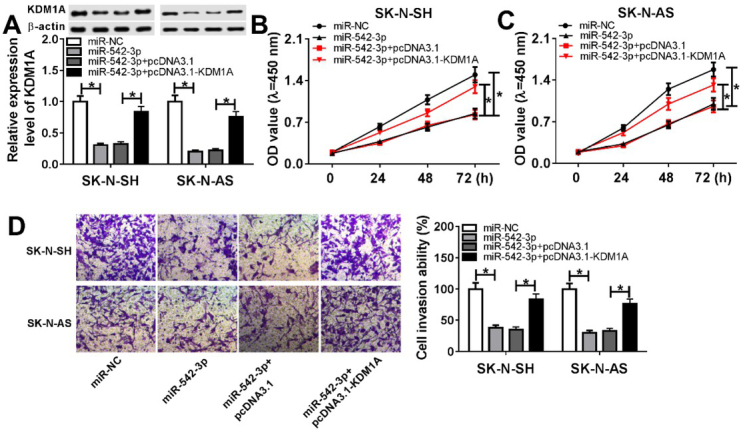
**The overexpression of KDM1A reversed the effect of miR-542-3p on neuroblastoma cells**. (A) Western blot assay was used to detect the protein level of KDM1A in SK-N-SH and SK-N-AS cells. (B and C) MTT assay was performed to assess cell proliferation in SK-N-SH cells (B) and SK-N-AS cells (C). (D) Transwell assay was carried out to measure cell invasion in SK-N-SH and SK-N-AS cells. *P<0.05. SK-N-SH and SK-N-AS cells were transfected with miR-NC, miR-542-3p, miR-542-3p + pcDNA3.1, or miR-542-3p + pcDNA3.1-KDM1A, respectively.

Based on the above results, it was speculated that miR-542-3p exerted function through inhibiting the expression of KDM1A. To verify this possibility, SK-N-SH and SK-N-AS cells were transfected with miR-542-3p or miR-542-3p + pcDNA3.1-KDM1A. Western blot assay demonstrated that the overexpression of miR-542-3p significantly downregulated the protein level of KDM1A, and the transfection with pcDNA3.1-KDM1A partly rescued the protein level of KDM1A (Figure 5A). Next, MTT assay suggested that cell proliferation was suppressed by the overexpression of miR-542-3p, and then rescued by the upregulation of KDM1A in SK-N-SH (Figure 5B) and SK-N-AS (Figure 5C) cells. Meanwhile, we also detected cell invasive ability, and found that the overexpression of KDM1A reversed the effect of miR-542-3p on cell invasion (Figure 5D). In summary, miR-542-3p inhibited cell proliferation and invasion through the downregulation of KDM1A in neuroblastoma.

### 3.6 MiR-542-3p downregulated ZNF346 expression through targeting ZNF346

**Figure 6:**
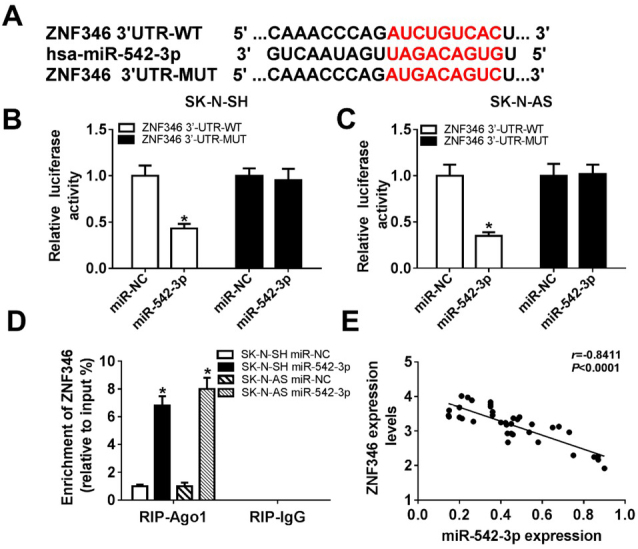
**The relationship between miR-542-3p and ZNF346 in neuroblastoma cells**. (A) The interaction between miR-542-3p and ZNF346 was predicted. (B and C) The luciferase activity of the cells transfected with ZNF346 3’-UTR-WT or ZNF346 3’-UTR-MUT and miR-543-3p or miR-NC was measured. (D) RIP assay was employed to confirm the interaction between miR-542-3p and ZNF346. (E) The relationship between miR-542-3p level and ZNF346 level was analyzed.

Using bioinformatics tool DIANA, we found that ZNF346 had a potential to target miR-542-3p (Figure 6A). Then, the dual luciferase reporter assay was employed to verify the interaction between miR-542-3p and ZNF346. As shown in Figure 6B and C, miR-542-3p overexpression significantly reduced the luciferase activity of ZNF346 3’-UTR-WT, but didn’t affect the luciferase activity of ZNF346 3’-UTR-MUT, revealing that ZNF346 interacted with miR-542-3p. Meanwhile, this interaction was also confirmed by RIP assay (Figure 6D). Next, the relationship between miR-542-3p level and ZNF346 level was evaluated. The results showed that ZNF346 level was negatively correlated with miR-542-3p level in neuroblastoma tissues (Figure 6E). These data suggested that ZNF346 was a target of miR-542-3p.

### 3.7 The overexpression of ZNF346 weakened effect of miR-542-3p on neuroblastoma cells

**Figure 7:**
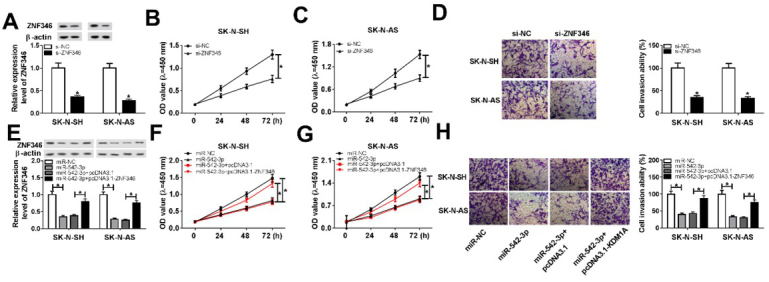
**The upregulation of ZNF346 impaired the effect of miR-542-3p on neuroblastoma cells**. (A) The expression of ZNF346 was investigated in SK-N-SH and SK-N-AS cells transfected with si-ZNF346 or si-NC. (B and C) Cell proliferation was assessed by MTT assay. (D) Cell invasion ability was explored using transwell assay. (E) ZNF346 level was determined in SK-N-SH and SK-N-AS cells transfected with miR-NC, miR-542-3p, miR-542-3p + pcDNA3.1, or miR-542-3p + pcDNA3.1-ZNF346 respectively. (F and G) MTT assay was carried out to measure cell proliferation. (H) Cell invasive ability was investigated by transwell assay. *P<0.05.

Through the analysis of ZNF346 function, we found that its knockdown suppressed cell proliferation and invasion (Figure 7A-D). Then, whether miR-542-3p regulating ZNF346 expression to modulate cellular phenotype was investigated. Firstly, SK-N-SH and SK-N-AS cells were transfected with miR-NC, miR-542-3p, miR-542-3p + pcDNA3.1, or miR-542-3p + pcDNA3.1-ZNF346, respectively. Western blot assay confirmed that ZNF346 expression was downregulated by miR-542-3p overexpression, and then upregulated by the transfection with pcDNA3.1-ZNF346 (Figure 7E). Then, cell proliferation ability was assessed using MTT assay. The results revealed that miR-542-3p overexpression inhibited cell proliferation, whereas this action was impaired by ZNF346 upregulation (Figure 7F and G). Furthermore, we analyzed cell migratory ability, and found that ZNF346 upregulation weakened the effect of miR-542-3p overexpression on cell migration in SK-N-SH and SK-N-AS cells (Figure 7H). Taken together, miR-542-3p mediated the growth of neuroblastoma cells via repressing ZNF346 expression.

### 3.8 MiR-542-3p negatively regulated the growth of tumor in neuroblastoma

**Figure 8:**
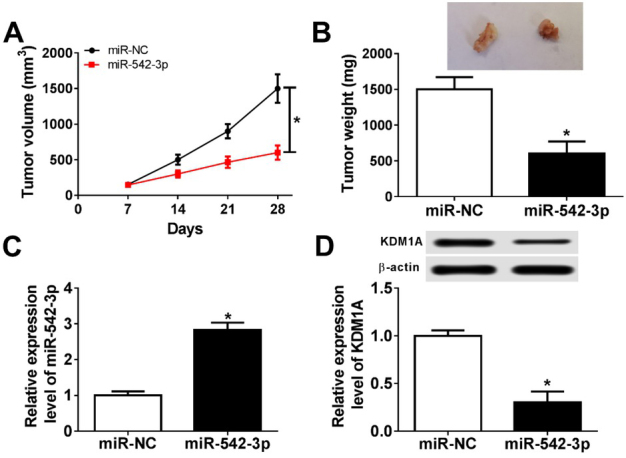
**The role of miR-542-3p in the progression of tumor development**. (A and B) Tumor volume (A) and weight (B) of female mice injected with miR-NC or miR-542-3p were calculated. (C and D) The mRNA level (C) and the protein level (D) of KDM1A were detected by qRT-PCR assay and western blot assay, respectively. *P<0.05.

To address the effect of miR-542-3p expression on neuroblastoma tumorigenesis *in vivo*, the cells transfected with miR-542-3p or empty vector were injected into female mice. Then, we measured tumor volume every 7 d, and found that the mice transfected with miR-542-3p exhibited smaller tumors than that transfected with empty vector (Figure 8A). Moreover, the rate of tumor growth in miR-542-3p mice was decreased with the growth of mice, and an opponent trend was observed in control mice.

In agreement with these results, tumor weight in miR-542 mice was smaller than that in the control mice (Figure 8B). Then, we detected the expression levels of miR-542-3p and KDM1A in the mice’s tumors. As expected, the expression of miR-542-3p was increased and the expression of KDM1A was decreased compared with the control group (Figure 8C and D). Therefore, the overexpression of miR-542-3p suppressed tumor growth of neuroblastoma *in vivo*.

## 4 Discussion

Neuroblastoma is a pediatric solid tumor with variable clinical outcomes among children [23]. MiRNAs exert function in many aspects of human cancers containing neuroblastomas. In this study, we confirmed that the overexpression of miR-542-3p suppressed cell proliferation, invasion, and tumor growth via the downregulation of KDM1A and ZNF346 in neuroblastomas. Our finding arises underlying mechanism and potential value for the treatment of neuroblastoma.

In recent years, many miRNAs were shown to modulate the development of neuroblastoma. For example, Han *et al*. proved that miR-223-3p promoted the growth as well as mobility through modulating forkhead box O1 (FOXO1) [27]. Liu *et al*. reported that miR-144 negatively the development and cisplatin resistance of pediatric neuroblastoma cells [28]. The previous reports suggested that miR-542-3p was downregulated in favorable histology, MYCN non-amplified neuroblastoma [29, 30]. In this study, we detected the expression level of miR-542-3p in neuroblastoma tissues and cells. The results demonstrated that miR-542-3p was lowly expressed in neuroblastoma tissues and cells, this was consistent with that. Moreover, miR-542, including miR-542-3p and miR-542-5p, was reported to inhibit cell proliferation, migration and invasion, as well as promoting cell apoptosis and death [14, 31]. In agreement with this data, our study confirmed that the upregulation of miR-542-3p significantly suppressed proliferation and invasion of neuroblastoma cells (SK-N-SH and SK-N-AS). Thus, it is concluded that miR-542-3p plays a negative role for neuroblastoma development.

In mammals, whether miR-542-3p interacts KDM1A or ZNF346 is unclear yet.

To explore the functional mechanism of miR-542-3p, its potential targets were searched.

Our data showed that miR-542-3p interacted with KDM1A and ZNF346. In general, miRNA negatively regulates expression of downstream genes by the induction of mRNA degradation or translational inhibition [32, 33]. Therefore, the overexpression of miRNA suppresses expression of its target gene. In this study, we analyzed the effect of miR-542-3p on KDM1A/ZNF346 expression. As expected, the levels of KDM1A and ZNF346 were positively modulated by miR-542-3p. So, we concluded that miR-542-3p decreased expression of KDM1A and ZNF346 by targeting their 3’-UTR.

In recent years, KDM1A and ZNF346 have been reported that they were closely related to neuroblastoma. Some scholars reported that the depletion of KDM1A increased the expression level of sestrin-2 (SESN2) and then resulted in the inhibition of mTOR complex 1 (mTORC1), which was good for sustainability of cellular homeostasis and was a protective mechanism against neurodegenerative diseases [32, 33]. Thus, KDM1A played pivotal roles in the progression of neuroblastoma development. Yang *et al* also confirmed that the upregulation of KDM1A suppressed cell proliferation, migration, and invasion in neuroblastoma [34]. Similarly, present evidence demonstrated that ZNF346 acted as an oncogene for the development of neuroblastoma [26]. Consistent with this data, the results in this study suggested that the knockdown of KDM1A and ZNF346 dramatically inhibited proliferation and invasion of neuroblastoma cells. Based on these results, we speculated that miR-542-3p inhibited neuroblastoma cell development through the downregulation of KDM1A and ZNF346. Thus, we enforced expression of KDM1A and ZNF346 under the condition of the overexpression of miR-542-3p. The results suggested that both KDM1A upregulation and ZNF346 overexpression restrained the function of miR-542-3p, confirming our conjecture. Therefore, miR-542-3p suppressed cell proliferation and invasion by regulating the levels of KDM1A and ZNF346 in neuroblastoma cells.

In general, miRNAs are involved in tumorigenesis in human cancers. For example, miR-181a/b was highly expressed, and promoted tumorigenesis in neuroblastoma [35], whereas the expression of miR-34a was decreased, and miR-34a suppressed tumorigenesis in neuroblastoma [36]. As a downregulated miRNA in neuroblastoma, miR-542-3p may play negative role for the formation of a tumor. Our data proved this possibility, miR-542-3p repressed tumor volume and weight in neuroblastoma.

Taken together, our data established an association between miR-542-3p and KDM1A/ZNF346, and suggested that miR-542-3p suppressed cell proliferation and invasion via targeting KDM1A and ZNF346 in neuroblastoma. These findings provides experimental evidence for further understanding of the molecular mechanism of neuroblastoma development and provide a theoretical basis for the therapy of neuroblastoma.
